# Costs and healthcare utilisation of patients with chronic kidney disease in Spain

**DOI:** 10.1186/s12913-021-06566-2

**Published:** 2021-06-01

**Authors:** Carlos Escobar, Beatriz Palacios, Unai Aranda, Margarita Capel, Antoni Sicras, Aram Sicras, Antonio Hormigo, Roberto Alcázar, Nicolás Manito, Manuel Botana

**Affiliations:** 1 University Hospital La Paz, Madrid, Spain; 2 AstraZeneca Farmacéutica Spain, SA Barcelona, Spain; 3 Health Economics and Outcomes Research, Atrys Health, Barcelona, Spain; 4 Primary care center Salud Puerta Blanca, Malaga, Spain; 5 University hospital Infanta Leonor, Madrid, Spain; 6 Hospital de Bellvitge, Hospitalet de Llobregat, Barcelona, Spain; 7 Hospital Universitario Lucus Augusti, Lugo, Spain

**Keywords:** Chronic kidney disease, Cost, DAPA-CKD, Healthcare, Hospitalization, Medication

## Abstract

**Background:**

Data about the impact of chronic kidney disease (CKD) on health care costs in Spain are scarce This study was aimed to evaluate cumulative costs and healthcare utilisation in CKD in Spain.

**Methods:**

Observational, retrospective, population-based study, which included adults who received care for CKD between 2015 and 2019. Healthcare and medication costs were summarized on a yearly basis starting from the index date (1st January 2015), and then cumulatively until 2019.

**Results:**

We identified 44,214 patients with CKD (year 2015: age 76.4 ± 14.3 years, 49.0% women, albumin-to-creatinine ratio 362.9 ± 176.8 mg/g, estimated glomerular filtration rate 48.7 ± 13.2 mL/min/1.73 m^2^). During the 2015–2019 period, cumulative CKD associated costs reached 14,728.4 Euros, being cardiovascular disease hospitalizations, particularly due to heart failure and CKD, responsible for 77.1% of costs. Total medication cost accounted for 6.6% of the total cost. There was a progressive decrease in cardiovascular disease hospital costs per year (from 2741.1 Euros in 2015 to 1.971.7 Euros in 2019). This also occurred with cardiovascular and diabetic medication costs, as well as with the proportion of hospitalizations and mortality. Costs and healthcare resources use were higher in the DAPA-CKD like population, but also decreased over time.

**Conclusions:**

Between 2015 and 2019, costs of patients with CKD in Spain were high, with cardiovascular hospitalizations as the key determinant. Medication costs were responsible for only a small proportion of total CKD costs. Improving CKD management, particularly with the use of cardiovascular and renal protective medications may be helpful to reduce CKD burden.

**Supplementary Information:**

The online version contains supplementary material available at 10.1186/s12913-021-06566-2.

## Introduction

Chronic kidney disease (CKD) is a common condition that affected nearly 700 million persons worldwide in 2017. However, these numbers are expected to rise due to the ageing of the population, and the increasing prevalence of hypertension and diabetes [1–3]. CKD markedly increases the risk of developing cardiovascular disease, particularly ischemic heart disease and heart failure [HF], as well as cardiovascular and all-cause death. In addition, CKD promotes the development of end-stage renal disease [2, 4]. Remarkably, the risk of adverse outcomes increases as renal function decreases or albuminuria develops [5].

The apropriate treatment of CKD patients has been associated with a reduction in the risk of developing cardiovascular and renal complications [6]. This is particularly true with the use of renin angiotensin system inhibitors, including angiotensin-converting enzyme inhibitors (ACEi) and angiotensin-receptor blockers (ARBs) [7, 8], and more recently, with the use of some sodium-glucose cotransporter-2 (SGLT-2) inhibitors [9, 10], even in the absence of type 2 diabetes (T2D) [9].

Of note, CKD represents a major and growing economic problem [3, 11, 12]. Increasing the knowledge about CKD-related costs is mandatory to ascertain how CKD management can be improved, leading to a significant decrease in CKD burden [5, 11–18]. Unfortunately, data about the impact of CKD on health care costs in Spain are scarce, and most importantly, not focused on a comprehensive approach [19, 20].

The aim of this study was to evaluate the cumulative costs and healthcare utilisation in CKD patients in Spain over the last 5 years, along with the epidemiological characterization of the population at index date (1st January 2015). This was also analyzed in a population who met the main inclusion criteria of the DAPA-CKD trial [9] (DAPA-CKD like population).

## Methods

This was an observational cohort study, comprising cross-sectional and longitudinal retrospective analyses using secondary data captured in electronic health records from seven Spanish regions, from the BIG-PAC® database. BIG-PAC® database included information from non-selected 1.7 million persons of primary health centers and referral hospitals within the Spanish national health system. Before export to BIG-PAC®, data were rigorously anonymized and dissociated, making not possible individual identification. As a result, it automatically collects information from routine practice, without requiring manual inputting. Previous studies have demonstrated its representativeness of the Spanish population [21, 22].

This database has been validated as an information source for studies of epidemiology, therapeutic adaptation and health/non-healthcare resource use and associated costs. It is representative of the Spanish population [21]. The study was approved by the Investigation Ethics Committee of Consorci Sanitari from Terrassa. Informed consent was waived by the same ethics committee that approved the study, as this was a secondary data study and data were fully anonymized and dissociated from patients. All methods were performed in accordance with the relevant guidelines and current regulations [21, 23].

The study population included all adult patients with at least one diagnostic code of CKD or having laboratory results meeting the definition of CKD prior to the index date (1st January 2015). CKD stages 1–5 were defined according to the estimated glomerular filtration rate (eGFR; estimated by the CKD-Epidemiology Collaboration equation) and the urine albumin-to-creatinine ratio (UACR), as follows: stage 1: eGFR ≥90 mL/min/1.73m^2^ and UACR ≥30 mg/g; stage 2: eGFR 60–89 mL/min/1.73m^2^ and UACR ≥30 mg/g; stage 3a: eGFR 45–59 mL/min/1.73m^2^; stage 3b: eGFR 30–44 mL/min/1.73m^2^; stage 4: eGFR 15–29 mL/min/1.73m^2^; stage 5: eGFR < 15 mL/min/1.73m^2^ [23, 24]. T2D was defined as all adult patients filling a prescription of any antidiabetic medication, having a T2D diagnostic code or HbA1c > 7% prior to index date, excluding type 1 diabetes. The DAPA-CKD like population included those adult patients, with or without T2D, but not type 1 diabetes, who had an eGFR of 25 to 75 mL/min/1.73 m^2^ and a UACR of 200 to 5000 mg/g, on stable treatment with ACEi or ARBs for at least 1 month [9].

Baseline characteristics for the overall CKD and DAPA-CKD like populations, including demographics, comorbidities and medications, were calculated at index date (1st January 2015) for the full group and by T2D status and CKD stage. The main comorbidities included myocardial infarction (MI), HF, atrial fibrillation (AF), stroke, peripheral artery disease (PAD), hyperkalemia and diabetes. A minimum of 1-year of data before index date was required. ICD-9 and ICD-10 codes (https://eciemaps.mscbs.gob.es) were considered for the diagnosis of comorbidities (supplementary Table 1). Treatments were recorded from the registries for dispensing medicines, according to the Anatomical Therapeutic Chemical Classification System (supplementary Table 1) [25]. Treatment for hypertension/HF (ACEi, ARBs, direct renin inhibitors, aldosterone antagonists, angiotensin receptor and neprilysin inhibition, beta blockers, diuretics, calcium channel blockers), antidiabetics (SGLT-2 inhibitors, metformin, sulfonylureas, dipeptidyl peptidase 4 [DPP-4] inhibitors, glucagon-like peptide-1 [GLP-1] receptor agonists, meglitinides, glitazones, acarbose, miglitol, insulin), antithrombotic therapy (warfarin, aspirin, P2Y12 receptor antagonists) and statins were recorded. Prescriptions were performed according to physicians´ criteria in routine practice [23].

Prevalence and incidence of CKD were also calculated at index date (1st January 2015). Incidence was calculated as all newly diagnosed patients during 2015 divided by the number of patients without CKD in the population at the beginning of 2015 and expressed in cases per 1000 patient-years. Prevalence was calculated as all patients with a CKD diagnosis at the end of 2015, divided by all individuals in the total population covered by the database at that time. The denominator included all individuals that were attended by any reason in the Spanish health care system in the previous 3 years to the index date (2012–2014). Mortality data were updated every month in the BIG-PAC database.

Costs were not taken from BIG-PAC database. Data were calculated using sources from the Spanish National Healthcare System of 2019 (supplementary Table 2) and used for the overall study period [21]. The healthcare resource use and costs and medication and procedure costs were summarized on a yearly basis starting from index date (1st January 2015), and then cumulatively until the end of the last year of follow-up (31st December 2019). All hospital visits (total and cardiovascular events), medical and emergency room visits, medication costs, and procedure costs (total, dialysis, kidney transplant) were included for the analysis of the annual direct healthcare costs [21]. Costs per patient were calculated every year. Patients who died during the follow-up had a cost of 0 allocated to the remaining duration of the study, whereas a patient leaving the database prior to data cut off was not included in the denominator for the time after leaving the database. No double counting occurred, as for each cost (i.e CKD hospitalizations) only that category was counted.

Annual indirect non-health costs included the number of days of productivity lost due to disability [21]. Rates were obtained from hospital accounting, except for medication and indirect costs, which were calculated as follows, respectively: a) medical prescriptions: according to the retail price per package at the time of dispensing [26]; b) costs for days of productivity lost: according to the mean interprofessional wage. The estimation of days off of work were obtained by the temporary work incapacity reported in primary care setting [27]. Hospital admission costs of cardiovascular events during follow-up were obtained taking into consideration the daily hospital rate and the number of hospital days per stay.

### Statistical analysis

Categorical variables were described by their absolute (n) and relative frequencies (%) and continuous variables by the mean and standard deviation. Categorical variables were compared with the Chi-square test and means by the t-student test. Analyses of health care cost were performed for the index date with 5 year of follow-up. The cumulative mean healthcare cost was estimated and presented on a yearly basis from the index date until last year of follow-up. Health care costs were presented per patient (mean cost). A level of statistical significance of 0.05 was applied in all the statistical tests. The data were analyzed using the statistical package SPSS v22.0 (SPSS Inc., Chicago, Illinois, USA) [21].

## Results

**Fig. 1:**
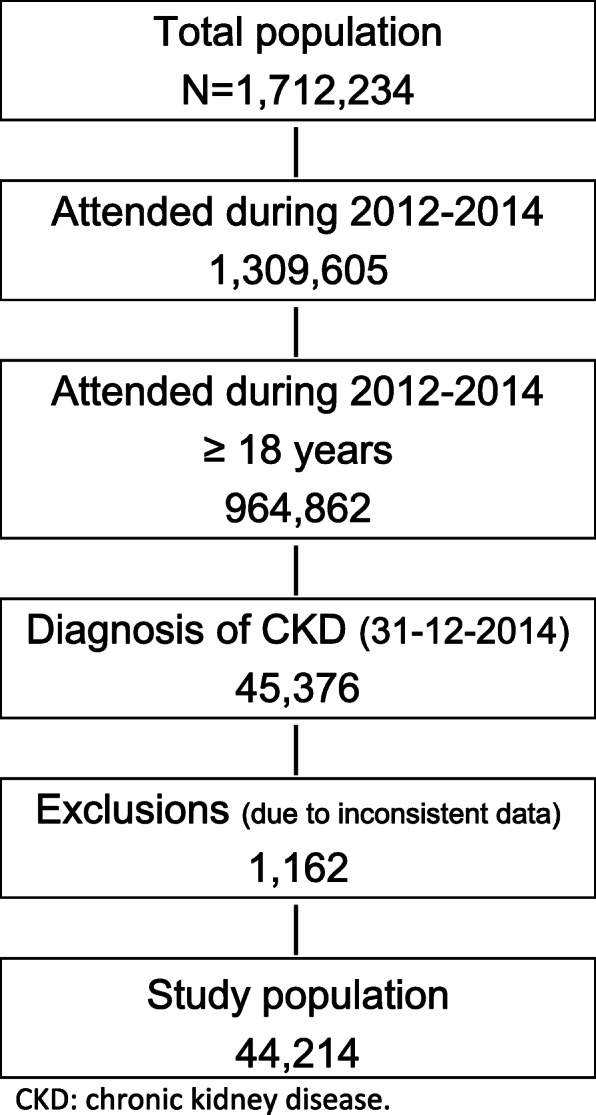
Flowchart costs population (2015). CKD: chronic kidney disease

Out of 1,7 million people included in the BIG-PAC® database in 2015, 1,3 were attended during the 2012–2014 period, of whom 964,862 were 18 years or older. At index date, 45.376 patients had CKD. As 1162 patients were excluded due to inconsistent data, 44,214 patients (97.4%) comprised the CKD study population (Fig. 1). Incidence at index date was 2.06 per 1000 patient-years and the prevalence was 4.90%.

The baseline clinical characteristics of the CKD population according to the presence of T2D and CKD stage are presented in Table 1. Overall, mean age was 76.4 ± 14.3 years, 49.0% of patients were women, mean UACR was 362.9 ± 176.8 mg/g and mean eGFR 48.7 ± 13.2 mL/min/1.73 m^2^. Overall, 20.0% of patients had a history of HF, 15.3% MI, and 10.5% stroke. With regard to treatments, 71.0% were taking renin angiotensin system (RAAS) inhibitors, but only 4.4% of patients at maximal doses. A total of 19,985 (45.2%) patients had T2D. Patients with T2D were younger (75.8 ± 14.0 vs 76.4 ± 14.1 years; *P* = 0.001), but UACR (391.3 ± 189.4 vs 347.4 ± 172.3 mg/g, *P* < 0.001), and HbA1c (7.7 ± 2.0 vs 6.2 ± 1.2%; *P* < 0.001) were higher and eGFR lower (47.5 ± 12.4 vs 49.6 ± 11.3 mL/min/1.73 m^2^, *P* < 0.001) compared to those without T2D. Moreover, comorbidities were more common among patients with T2D. In addition, more T2D patients were taking RAAS inhibitors (82.2% vs 61.7%; *P* < 0.001). Overall, 71.0% of patients had stage ≥3 CKD. Age increased as renal function worsened (from 69.8 ± 14.7 years in patients with stage 1 CKD to 79.8 ± 14.6 years among stage 5 CKD patients; *P* < 0.001), as well as UACR (from 106.8 ± 49.8 mg/g to 1642.3 ± 769.2 mg/g; *P* < 0.001) and the proportion of patients treated with RAAS inhibitors (from 66.5 to 72.6%; *P* < 0.001). Similarly, comorbidities increased as renal function decreased (Table 1).

**Table 1:** Baseline clinical characteristics of the CKD population at index date (1st January 2015) and according to the presence of type 2 diabetes and CKD stage

	Diabetes status			CKD stage													Total (***n*** = 44,214; 100%)
	Non T2D (***n*** = 24,229; 54.8%)	T2D (***n*** = 19,985; 45.2%)	p	Stage 1 (***n*** = 2159; 4.9%)	Stage 2 (***n*** = 7386;16.7%)	P_**2 vs 1**_	Stage 3a (***n*** = 14,065; 31.8%)	P_**3a vs 1**_	Stage 3b (***n*** = 12,203; 27.6%)	P_**3b vs 1**_	Stage 4 (***n*** = 3537; 8.0%)	P_**4 vs 1**_	Stage 5 (***n*** = 1592; 3.6%)	P_**5 vs 1**_	Unspecified (***n*** = 3272; 7.4%)	P_**Unsp. vs 1**_	
**Biodemographic data**																	
Age, years	76.4 ± 14.1	75.8 ± 14.0	0.001	69.8 ± 14.7	74.5 ± 13.9	< 0.001	76.8 ± 14.2	< 0.001	78.1 ± 14.3	< 0.001	79.2 ± 14.5	< 0.001	79.8 ± 14.6	< 0.001	65.1 ± 11.4	< 0.001	76.4 ± 14.3
Sex, Female, n (%)	11,892 (49.1)	9773 (48.9)	< 0.001	1105 (51.2)	3545 (48.0)	< 0.001	6779 (48.2)	< 0.001	5992 (49.1)	0.072	1680 (47.5)	< 0.001	876 (55.0)	< 0.001	1688 (51.6)	0.773	21,665 (49.0)
**Physical examination and laboratory tests**																	
BMI, Kg/m^2^	28.4 ± 5.2	29.3 ± 5.0	< 0.001	29.3 ± 5.0	29.2 ± 5.0	0.307	28.6 ± 5.2	< 0.001	28.5 ± 5.1	< 0.001	28.1 ± 5.0	< 0.001	27.8 ± 4.9	< 0.001	29.6 ± 5.1	0.007	28.7 ± 5.1
SBP, mmHg	136.2 ± 20.2	138.3 ± 20.3	< 0.001	135.9 ± 20.1	138.2 ± 20.6	< 0.001	138.3 ± 20.6	0.194	138.6 ± 20.4	0.148	137.5 ± 20.7	0.392	135.2 ± 20.5	0.791	137.8 ± 20.0	0.392	137.1 ± 20.2
UACR, mg/g	347.4 ± 172.3	391.3 ± 189.4	< 0.001	106.8 ± 49.8	126.9 ± 53.5	< 0.001	250.3 ± 120.1	< 0.001	252.1 ± 120.9	< 0.001	1602.4 ± 782.6	< 0.001	1642.3 ± 769.2	< 0.001	132.7 ± 60.2	< 0.001	362.9 ± 176.8
UACR A1, n (%)	137 (0.56)	80 (0.4)	< 0.001	0	0	–	0	–	0	–	0	–	0	–	217 (6.6)	–	217 (0.5)
UACR A2, n (%)	16,493 (68.1)	9772 (48.9)	< 0.001	2159 (100)	7386 (100)	–	8650 (61.5)	< 0.001	7480 (61.3)	< 0.001	432 (12.2)	< 0.001	53 (3.3)	< 0.001	105 (3.2)	< 0.001	26,265 (64.5)
UACR A3, n (%)	7599 (31.4)	10,133 (50.7)	< 0.001	0	0	–	5415 (38.5)	< 0.001	4723 (38.7)	< 0.001	3105 (87.8)	< 0.001	1539 (96.7)	< 0.001	2950 (90.2)	< 0.001	17,729 (35.0)
eGFR^a^	49.6 ± 11.3	47.5 ± 12.4	< 0.001	93.8 ± 4.7	75.1 ± 5.0	< 0.001	52.2 ± 4.9	< 0.001	37.2 ± 5.0	< 0.001	21.9 ± 4.5	< 0.001	8.7 ± 4.3	< 0.001	–	–	48.7 ± 13.2
eGFR ≥90^a^,n (%)	1324 (5.5)	835 (4.2)	< 0.001	2159 (100)	0	–	0	–	0	–	0	–	0	–	0	–	2159 (4.9)
eGFR 60–89^a^, n (%)	4216 (17.4)	3170 (15.9)	< 0.001	0	7386 (100)	–	0	–	0	–	0	–	0	–	0	–	7386 (16.7)
eGFR 45–59^a^, n (%)	7931 (32.7)	6134 (30.7)	< 0.001	0	0	–	14,065 (100)	–	0	–	0	–	0	–	0	–	14,065 (31.8)
eGFR 30–44^a^, n (%)	6572 (27.1)	5631 (28.2)	< 0.001	0	0	–	0	–	12,203 (100)	–	0	–	0	–	0	–	12,203 (27.6)
eGFR 15–29^a^, n (%)	1730 (7.1)	1807 (9.0)	< 0.001	0	0	–	0	–	0	–	3537 (100)	–	0	–	0	–	3537 (8.0)
eGFR < 15^a^, n (%)	644 (2.7)	948 (4.7)	< 0.001	0	0	–	0	–	0	–	0		1592 (100)	–	0	–	1592 (3.6)
HbA1c. %	6.2 ± 1.2	7.7 ± 2.0	< 0.001	6.4 ± 1.3	6.6 ± 1.4	0.001	6.9 ± 1.6	0.001	6.8 ± 1.5	< 0.001	6.9 ± 1.6	0.001	7.0 ± 1.5	0.001	7.0 ± 1.6	0.389	6.9 ± 1.6
Creatinine. g/dL	1.3 ± 0.4	1.3 ± 0.5	0.759	0.6 ± 0.3	0.9 ± 0.4	< 0.001	1.1 ± 0.5	< 0.001	1.5 ± 0.8	< 0.001	2.2 ± 0.8	< 0.001	0.6 ± 0.2	0.999	1.0 ± 0.3	< 0.001	1.3 ± 0.6
Uric acid. g/dL	6.0 ± 2.0	7.1 ± 1.2	< 0.001	6.6 ± 1.5	6.8 ± 1.6	0.041	6.7 ± 1.6	0.001	6.6 ± 1.5	0.999	6.7 ± 1.6	0.284	6.6 ± 1.6	0.999	6.7 ± 1.7	0.229	6.6 ± 1.6
**Comorbidities, n (%)**																	
CKD stage 1	1324 (5.5)	835 (4.2)	< 0.001	2159 (100)	0	< 0.001	0	–	0	–	0	–	0	–	0	–	2159 (4.9)
CKD stage 2	4216 (17.4)	3170 (15.9)	< 0.001	0	7386 (100)	< 0.001	0	–	0	–	0	–	0	–	0	–	7386 (16.7)
CKD stage 3a	7931 (32.7)	6134 (30.7)	< 0.001	0	0	–	14,065 (100)	–	0	–	0	–	0	–	0	–	14,065 (31.8)
CKD stage 3b	6572 (27.1)	5631 (28.2)	< 0.001	0	0	–	0	–	12,203 (100)	–	0	–	0	–	0	–	12,203 (27.6)
CKD stage 4	1730 (7.1)	1807 (9.0)	< 0.001	0	0	–	0	–	0	–	3537 (100)	–	0	–	0	–	3537 (8.0)
CKD stage 5	644 (2.7)	948 (4.7)	< 0.001	0	0	–	0	–	0	–	0	–	1592 (100)	–	0	–	1592 (3.6)
CKD not staged	1812 (7.5)	1460 (7.0)	< 0.001	0	0	–	0	–	0	–	0	–	0	–	3272 (100)	–	3272 (7.4)
CKD unspecified	6844 (28.2)	799 (4.0)	< 0.001	319 (14.8)	1079 (14.6)	0.629	2411 (17.1)	< 0.001	2173 (17.8)	0.001	508 (14.4)	0.678	237 (14.9)	0.932	916 (28.0)	< 0.001	7643 (17.3)
Dialysis	226 (0.9)	430 (3.2)	< 0.001	0	0		0	–	0		0	–	656 (41.2)	–	0	–	656 (1.5)
CVD	3616 (14.9)	4579 (22.9)	< 0.001	296 (13.7)	1230 (16.7)	< 0.001	2643 (18.8)	< 0.001	2295 (18.8)	< 0.001	597 (16.9)	< 0.001	386 (24.3)	< 0.001	748 (22.9)	< 0.001	8195 (18.5)
Myocardial infarction	3081 (12.7)	3671 (18.4)	< 0.001	253 (11.7)	916 (12.4)	0.383	2255 (16.0)	< 0.001	1920 (15.7)	< 0.001	575 (16.3)	< 0.001	274 (17.2)	< 0.001	559 (17.1)	< 0.001	6752 (15.3)
Heart failure	4078 (16.8)	4782 (23.9)	< 0.001	253 (11.7)	998 (13.5)	< 0.001	2768 (19.7)	< 0.001	2601 (21.3)	< 0.001	831 (23.5)	< 0.001	453 (28.4)	< 0.001	956 (29.2)	< 0.001	8860 (20.0)
Stroke	2136 (8.8)	2492 (12.5)	< 0.001	133 (6.2)	716 (9.7)	< 0.001	1218 (8.7)	< 0.001	1536 (12.6)	< 0.001	398 (11.3)	< 0.001	216 (13.6)	< 0.001	411 (12.6)	< 0.001	4628 (10.5)
Atrial Fibrillation	3508 (14.5)	3306 (16.5)	< 0.001	253 (11.7)	971 (13.2)	0.067	2374 (16.9)	< 0.001	2000 (16.4)	< 0.001	609 (17.2)	< 0.001	278 (17.5)	< 0.001	329 (10.1)	0.062	6814 (15.4)
Peripheral artery disease	1003 (4.1)	921 (4.6)	< 0.001	94 (4.4)	295 (4.0)	0.409	481 (3.4)	0.019	653 (5.4)	0.055	171 (4.8)	0.487	81 (5.1)	0.317	149 (4.6)	0.728	1924 (4.4)
Diabetes	775 (3.2)	19,985 (100)	< 0.001	933 (43.2)	3483 (47.2)	< 0.001	6604 (47.0)	< 0.001	5890 (48.3)	< 0.001	1643 (46.5)	0.015	712 (44.7)	0.360	1495 (45.7)	< 0.001	20,760 (47.0)
**Medications, n (%)**																	
**Antihypertensives**	17,518 (72.3)	17,346 (86.8)	< 0.001	1625 (75.3)	5628 (76.2)	0.389	10,844 (77.1)	0.065	9737 (79.8)	< 0.001	2975 (84.1)	< 0.001	1345 (84.5)	< 0.001	2849 (87.1)	< 0.001	33,337 (75.4)
RAAS inhibitors	14,944 (61.7)	16,427 (82.2)	< 0.001	1436 (66.5)	5449 (73.8)	< 0.001	10,267 (73.0)	< 0.001	8490 (69.6)	< 0.001	2530 (71.5)	< 0.001	1156 (72.6)	< 0.001	2043 (62.4)	< 0.001	31,371 (71.0)
ACEi	7198 (29.7)	6789 (34.0)	< 0.001	711 (32.9)	2539 (34.4)	0.196	4286 (30.5)	< 0.001	3467 (28.4)	< 0.001	1265 (35.8)	0.010	592 (37.2)	< 0.001	1127 (34.4)	0.253	13,987 (31.6)
ACEi at maximal doses	339 (1.4)	401 (2.0)	< 0.001	88 (4.1)	28 (0.4)	< 0.001	181 (1.3)	< 0.001	475 (3.9)	0.659	30 (0.8)	< 0.001	27 (1.7)	< 0.001	33 (1.0)	< 0.001	740 (1.7)
ARBs	8509 (35.1)	10,960 (54.8)	< 0.001	874 (40.5)	2953 (40.0)	0.677	6446 (45.8)	< 0.001	5690 (46.6)	< 0.001	1675 (47.4)	< 0.001	684 (43.0)	< 0.001	1147 (35.1)	< 0.001	19,469 (44.0)
ARBs at maximal doses	465 (1.9)	736 (3.7)	< 0.001	22 (1.0)	0	–	660 (4.7)	< 0.001	393 (3.2)	< 0.001	110 (3.1)	< 0.001	16 (1.0)	0.999	0	–	1201 (2.7)
Aldosterone antagonists	1471 (6.1)	1469 (7.4)	< 0.001	155 (7.2)	325 (4.4)	< 0.001	858 (6.1)	0.049	1031 (8.4)	0.061	288 (8.1)	0.218	151 (9.5)	< 0.001	132 (4.0)	< 0.001	2940 (6.6)
Direct renin inhibitors	170 (0.7)	118 (0.6)	< 0.001	8 (0.4)	26 (0.4)	0.999	80 (0.6)	0.252	84 (0.7)	0.111	30 (0.8)	0.068	12 (0.8)	0.108	48 (1.5)	< 0.001	288 (0.7)
ARNI	1124 (4.6)	2581 (12.9)	< 0.001	202 (9.4)	631 (8.5)	0.192	1374 (9.8)	0.560	914 (7.5)	0.002	231 (6.5)	0.001	143 (9.0)	0.676	210 (6.4)	< 0.001	3705 (8.4)
Beta blockers	8315 (34.3)	7656 (38.3)	< 0.001	598 (27.7)	2370 (32.1)	< 0.001	5186 (36.9)	< 0.001	4682 (38.4)	< 0.001	1434 (40.5)	< 0.001	685 (43.0)	< 0.001	1016 (31.1)	< 0.001	15,971 (36.1)
Diuretics	8957 (37.0)	9260 (46.3)	< 0.001	611 (28.3)	2755 (37.3)	< 0.001	5802 (41.3)	< 0.001	5607 (45.9)	< 0.001	1592 (45.0)	< 0.001	785 (49.3)	< 0.001	1065 (32.5)	< 0.001	18,217 (41.2)
Thiazide diuretics	906 (3.7)	1347 (6.7)	< 0.001	33 (1.5)	420 (5.7)	< 0.001	715 (5.1)	< 0.001	742 (6.1)	< 0.001	174 (4.9)	< 0.001	39 (2.4)	0.045	130 (4.0)	< 0.001	2253 (5.1)
Loop diuretics	7986 (33.0)	7884 (39.4)	< 0.001	511 (23.7)	2228 (30.2)	< 0.001	5063 (36.0)	< 0.001	4852 (39.8)	< 0.001	1552 (43.9)	< 0.001	724 (45.5)	< 0.001	940 (28.7)	< 0.001	15,870 (35.9)
Potassium sparing diuretics	1112 (4.6)	2457 (12.3)	< 0.001	110 (5.1)	538 (7.3)	< 0.001	1387 (9.9)	< 0.001	1025 (8.4)	< 0.001	210 (5.9)	0.203	175 (11.0)	< 0.001	124 (3.8)	0.021	3569 (8.1)
CCB	6280 (25.9)	7913 (39.6)	< 0.001	596 (27.6)	2457 (33.3)	< 0.001	4647 (33.0)	< 0.001	3761 (30.8)	< 0.001	1165 (32.9)	< 0.001	521 (32.7)	< 0.001	1046 (32.0)	< 0.001	14,193 (32.1)
Dihydropyridines	6300 (26.0)	6803 (34.0)	< 0.001	613 (28.4)	2225 (30.1)	< 0.001	3910 (27.8)	0.563	3716 (30.5)	0.050	1135 (32.1)	< 0.001	490 (30.8)	< 0.001	1014 (31.0)	< 0.001	13,103 (29.6)
Non-dihydropyridines	1265 (5.2)	397 (2.0)	< 0.001	55 (2.5)	246 (3.3)	0.060	768 (5.5)	< 0.001	210 (1.7)	0.010	198 (5.6)	< 0.001	39 (2.4)	0.845	146 (4.5)	< 0.001	1662 (3.8)
**Antidiabetics**	218 (0.9)	16,685 (83.5)	< 0.001	814 (37.7)	2968 (40.2)	< 0.001	4965 (35.3)	< 0.001	4596 (37.7)	0.999	1552 (43.9)	< 0.001	816 (51.3)	< 0.001	1192 (36.4)	0.331	16,903 (38.2)
Metformin	0	9645 (48.3)	< 0.001	564 (26.1)	1854 (25.1)	0.347	2768 (19.7)	< 0.001	2454 (20.1)	< 0.001	1062 (30.0)	< 0.001	562 (35.3)	< 0.001	381 (11.6)	< 0.001	9645 (21.8)
Sulfonylurea	0	2260 (11.3)	< 0.001	122 (5.7)	477 (6.5)	0.179	603 (4.3)	0.003	602 (4.9)	0.117	230 (6.5)	0.224	144 (9.0)	< 0.001	82 (2.5)	< 0.001	2260 (5.1)
DPP4 inhibitors	0	7682 (38.4)	< 0.001	323 (15.0)	1288 (17.4)	< 0.001	2607 (18.5)	< 0.001	2178 (17.8)	< 0.001	556 (15.7)	0.478	298 (18.7)	< 0.001	432 (13.2)	< 0.001	7682 (17.4)
SGLT-2 inhibitors	0	401 (2.0)	< 0.001	23 (1.1)	44 (0.6)	0.015	82 (0.6)	0.008	57 (0.5)	0.001	135 (3.8)	< 0.001	44 (2.8)	< 0.001	16 (0.5)	0.012	401 (0.9)
GLP-1 receptor agonists	0	695 (3.5)	< 0.001	47 (2.2)	104 (1.4)	0.001	121 (0.9)	0.001	223 (1.8)	0.205	95 (2.7)	0.242	53 (3.3)	0.039	52 (1.6)	0.107	695 (1.6)
Metiglinides	0	2992 (15.0)	< 0.001	89 (4.1)	390 (5.3)	< 0.001	1067 (7.6)	< 0.001	955 (7.8)	< 0.001	164 (4.6)	0.373	69 (4.3)	0.762	258 (7.9)	< 0.001	2992 (6.8)
Glitazones	0	259 (1.3)	< 0.001	17 (0.8)	5 (0.1)	0.114	52 (0.4)	0.010	68 (0.6)	0.279	58 (1.6)	0.010	33 (2.1)	0.001	26 (0.8)	0.999	259 (0.6)
Acarbose	0	314 (1.6)	< 0.001	48 (2.2)	53 (0.7)	< 0.001	36 (0.3)	< 0.001	75 (0.6)	0.001	65 (1.8)	0.290	35 (2.2)	0.999	2 (0.1)	< 0.001	314 (0.7)
Insulin	218 (0.9)	4246 (21.2)	< 0.001	155 (7.2)	719 (9.7)	< 0.001	1411 (10.0)	< 0.001	1256 (10.3)	< 0.001	376 (10.6)	< 0.001	213 (13.4)	< 0.001	334 (10.2)	< 0.001	4464 (10.1)
Statins	11,160 (46.1)	12,798 (64.0)	< 0.001	1140 (52.8)	4014 (54.3)	0.218	7734 (55.0)	< 0.001	6685 (54.8)	0.006	1906 (53.9)	0.419	879 (55.2)	< 0.001	1600 (48.9)	< 0.001	23,958 (54.2)
Warfarin	2849 (11.8)	3092 (15.5)	< 0.001	189 (8.8)	921 (12.5)	< 0.001	1972 (14.0)	< 0.001	1728 (14.2)	< 0.001	504 (14.2)	< 0.001	205 (12.9)	< 0.001	422 (12.9)	< 0.001	5941 (13.4)
Low dose aspirin	6045 (25.0)	5499 (27.5)	< 0.001	446 (20.7)	1792 (24.3)	< 0.001	3847 (27.4)	< 0.001	3198 (26.2)	< 0.001	1026 (29.0)	< 0.001	445 (28.0)	< 0.001	790 (24.1)	< 0.001	11,544 (26.1)
Receptor P2Y12 antagonists	872 (3.6)	1998 (10.0)	< 0.001	110 (5.1)	469 (6.3)	0.039	774 (5.5)	0.446	919 (7.5)	< 0.001	274 (7.7)	< 0.001	171 (10.7)	< 0.001	153 (4.7)	0.502	2870 (6.5)

*ACEi* angiotensin-converting enzyme inhibitors, *ARBs* angiotensin receptor blockers, *ARNI* angiotensin receptor and neprilysin inhibition, *BMI* body mass index, *CCB* Calcium channel blockers; CVD: cardiovascular disease, *CKD* chronic kidney disease, *DPP4* dipeptidyl peptidase 4, *eGFR* estimated glomerular filtration rate, ^a^ mL/min/1.73 m^2^, *GLP-1* glucagon-like peptide-1, *PAD* peripheral artery disease, *RAAS* renin angiotensin system, *SBP* systolic blood pressure, *SGLT-2* sodium-glucose Cotransporter-2, *UACR* Urine albumin-to-Creatinine Ratio

**Fig. 2:**
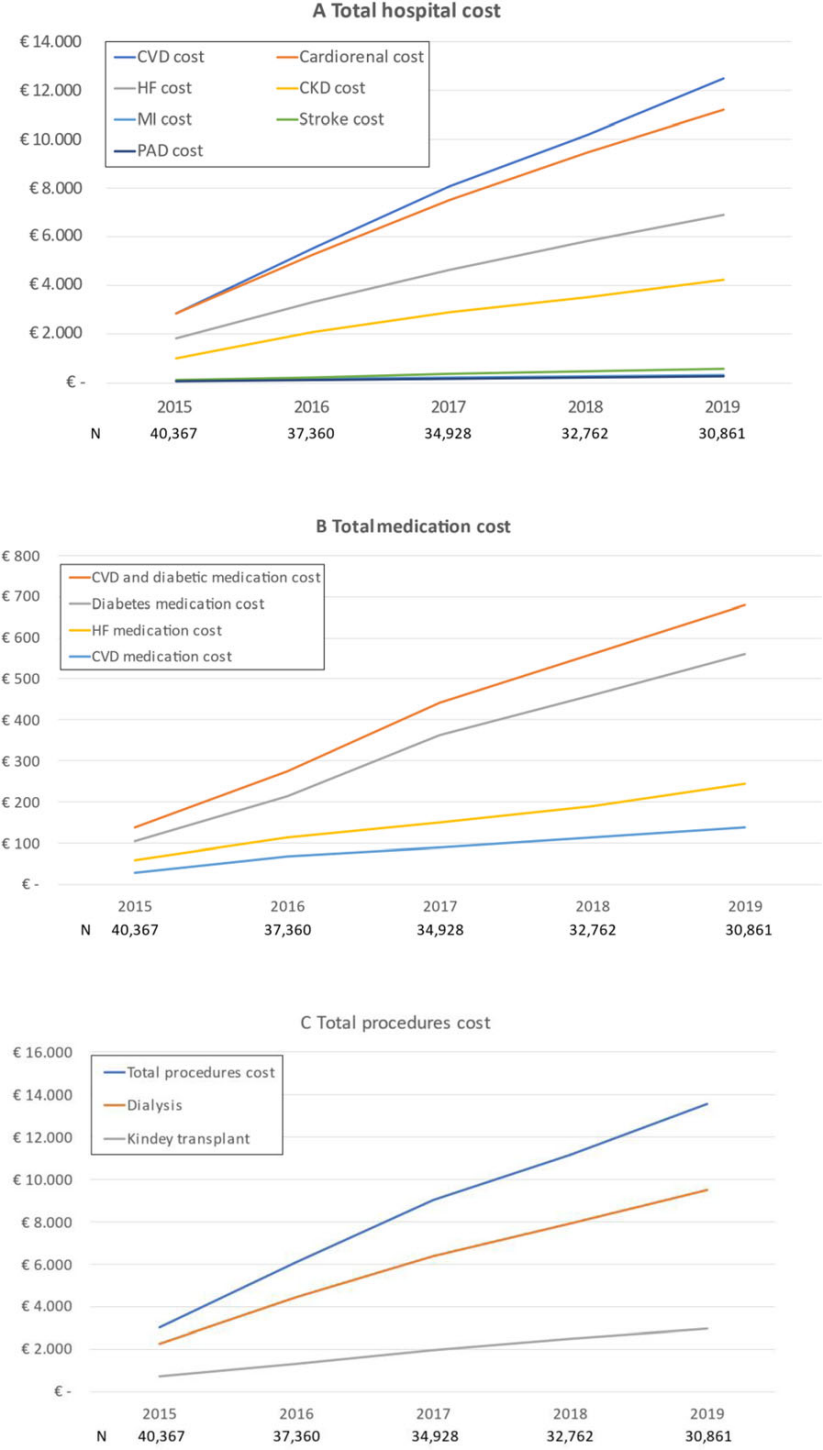
Patient cumulative hospital (**A**), medication (**B**) and procedures (**C**) mean costs*. *In Euros. CVD: cardiovascular disease; HF: heart failure; CKD: chronic kidney disease; MI: myocardial infarction; PAD: peripheral artery disease. Cardiorenal costs include HF and CKD costs

Patient hospital mean costs per year are presented in Table 2. From 2015 to 2019 there was a progressive decrease in cardiovascular disease hospital cost per patient year (from 2741.1 to 1971.7 Euros) and patient cumulative cardiovascular disease hospital mean cost reached 11,349.2 Euros in 2019 (supplementary Table 3 and Fig. 2a). The great burden of hospital cost was due to cardiovascular hospitalizations, particularly HF and CKD. Regarding medications, from 2015 to 2019, diabetes drugs mean cost decreased from 102.71 to 89.99 Euros per patient and year, but HF medication mean cost slightly increased from 50.68 to 53.04 Euros, respectively (Table 2). The cumulative mean cost of diabetes and HF medications reached 503.9 and 220.6 Euros, respectively, in 2019 (supplementary Table 3 and Fig. 2b). Dialysis cost decreased from 2328.8 to 1624.2 Euros, respectively (cumulative cost of 9602.9 Euros) and kidney transplant from 655.7 to 465.2 Euros, respectively (cumulative cost of 2701.8 Euros) (supplementary Table 3 and Fig. 2c).

**Table 2:** Patients hospital mean cost per year^a^

	2015		2016		2017		2018		2019		Cumulative cost in 2019
	mean	SD	mean	SD	mean	SD	mean	SD	mean	SD	
**Total hospital cost**											
CVD	2741.1	5097.5	2452.6	5049.5	2308.3	5001.4	1875.5	4953.3	1971.7	4953.3	11,349.2
Cardiorenal	2500.3	4924.3	2250.7	4654.3	2105.9	4180.7	1685.4	3412.9	1766.7	3935.4	10,309.0
HF	1514.3	3602.4	1341.9	3160.5	1283.9	3023.9	1115.0	2520.9	1012.6	2426.7	6267.7
CKD	986.0	4.044.9	908.7	4026.2	822.0	3860.2	570.4	2866.6	754.2	3410.9	4041.3
MI	74.3	732.7	61.6	589.2	55.0	516.1	55.3	560.2	65.4	596.4	311.5
Stroke	111.7	784.8	99.4	754.1	105.4	792.1	94.6	732.0	99.8	749.0	510.9
PAD	54.9	658.9	41.0	506.8	42.0	544.6	40.2	521.5	39.7	551.9	217.8
**Medication cost**											
Total medication	181.80	384.70	178.83	382.2	180.97	352.0	142.66	249.7	167.76	323.0	852.0
Diabetes medication	102.71	341.10	99.81	348.0	128.51	443.6	82.84	291.7	89.99	291.5	503.9
HF medication	50.68	76.76	47.34	72.4	31.88	53.2	37.71	60.4	53.04	93.4	220.6
CVD medication	28.41	62.40	31.68	71.7	20.58	46.1	22.11	45.6	24.73	53.0	127.5
**Procedure costs**											
Total procedures	2984.5	23,547.4	2657.5	21,616.1	2516.5	20,106.6	2056.8	15,954.3	2089.4	16,415.2	12,304.7
Dialysis	2328.8	22,021.0	2081.3	19,672.1	1965.2	18,523.3	1603.4	15,265.7	1624.2	15,465.8	9602.9
Kidney transplant	655.7	4720.9	576.2	4026.3	551.3	3898.3	453.4	3.267.1	465.2	3402.9	2701.8

*CVD* cardiovascular disease, *HF* heart failure, *CKD* chronic kidney disease, *MI* myocardial infarction, *PAD* peripheral artery disease

^a^In Euros. Cardiorenal disease includes HF and CKD

The health resources use for each year is shown in Table 3. The proportion of hospitalized patients decreased from 27.6% in 2015 to 21.2% in 2019; *P* < 0.001, the days for hospitalized patients from 16.4 to 11.2 days; *P* < 0.001, and the proportion of patients that died from 8.7 to 4.3%; *P* < 0.001, respectively. Total health-related cost decreased from 3561 Euros in 2015 to 2493 Euros in 2019. Including indirect costs, total cumulative patient mean costs reached 14,728.4 Euros in 2019; 651,203,550.3 Euros per total CKD population (Table 4).

**Table 3:** Health resources use for each year per patient

	2015		2016		P_**2016–2015**_	2017		P_**2017–2015**_	2018		P_**2018–2015**_	2019		P_**2019–2015**_	Total	
	mean	SD	mean	SD		mean	SD		mean	SD		mean	SD		mean	SD
Primary care visits	12.4	14.8	10.6	12.3	< 0.001	9.2	10.4	< 0.001	8.4	9.6	< 0.001	7.5	8.3	< 0.001	48.1	55.8
Specialized care visits	1.9	4.1	1.1	4.3	< 0.001	1.0	4.2	< 0.001	0.9	4.1	< 0.001	0.7	3.9	< 0.001	5.6	7.2
Emergency room visits	0.7	2.3	0.6	2.6	< 0.001	0.5	2.1	< 0.001	0.5	2.1	< 0.001	0.4	1.9	< 0.001	2.7	4.5
Laboratory requests	0.8	1.2	0.7	1.5	< 0.001	0.7	1.5	< 0.001	0.6	1.4	< 0.001	0.6	1.3	< 0.001	3.4	5.1
Radiology and other tests	0.6	1.3	0.6	1.3	–	0.6	1.4	–	0.7	1.5	< 0.001	0.7	1.5	< 0.001	3.2	4.2
Hospitalization																
- Days (all patients)	5.7	10.6	5.1	10.5	< 0.001	4.8	10.4	< 0.001	3.9	10.3	< 0.001	4.1	10.3	< 0.001	23.6	37.6
- Hospitalized patients, n (%)	12,203 (27.6)		10,832 (24.5)		< 0.001	10,081 (22.8)		< 0.001	9727 (22.0)		< 0.001	9373 (21.2)		< 0.001	19,108 (43.2)	
- Days (for patients hospitalized)	16.4	10.6	16.5	10.6	0.161	16.7	10.70	< 0.001	16.8	11.0	< 0.001	17.0	11.2	< 0.001	4.2	23.6
- Frequency of hospitalization, n (%)																
0	32,011 (72.4)		32,497 (73.5)		< 0.001	33,515 (75.8)		< 0.001	34,089 (77.1)		< 0.001	34,398 (77.8)		< 0.001	25,106 (57.0)	
1	10,302 (23.3)		9904 (22.4)		0.001	8754 (19.8)		< 0.001	8091 (18.3)		< 0.001	7783 (17.6)		< 0.001	6986 (15.8)	
2	1636 (3.7)		1680 (3.8)		0.434	1724 (3.9)		0.12	1769 (4.0)		0.02	1724 (3.9)		0.12	5261 (11.9)	
3+	265 (0.6)		133 (0.3)		< 0.001	221 (0.5)		0.044	265 (0.6)		0.999	309 (0.7)		0.064	6862 (15.5)	
Disability																
Days of disability	0.3	4.2	0.3	4.5	–	0.4	5.6	0.003	0.4	5.9	0.004	0.4	5.9	0.004	1.9	17.5
Average days of sick leave (disability only)	41.3	42.3	42.4	43.6	< 0.001	45.3	46.5	< 0.001	44.2	47.1	< 0.001	46.1	47.6	< 0.001	60.3	63.4
Patients with disability, n (%)	354 (0.8)		354 (0.8)		–	398 (0.9)		0.105	442 (1.0)		0.002	398 (0.9)		0.105	1326 (3.0)	
Mortality, n (%)	3847 (8.7)		3007 (6.8)		< 0.001	2432 (5.5)		< 0.001	2166 (4.9)		< 0.001	1901 (4.3)		< 0.001	13,353 (30.2)	
Patients alive at the end of the year, n	40,367		37,360		–	34,928		–	32,762		–	30,861		–	–

**Table 4:** Total mean cost for year and cumulative cost in 2019^a^ per patient

	2015		2016		2017		2018		2019		Cumulative cost in 2019
	mean	SD	mean	SD	mean	SD	mean	SD	mean	SD	
Primary care visits	300.1	358.2	256.5	297.7	222.6	251.7	203.3	232.3	181.5	200.9	1164.0
Laboratory requests	25.8	38.8	22.6	48.5	22.6	48.5	19.4	45.2	19.4	42.0	109.8
Radiology and other tests	17.1	37.1	17.1	37.1	17.1	39.9	20.0	42.8	20.0	42.8	91.2
Specialized visits	179.6	387.5	104.0	406.4	94.5	396.9	85.1	387.5	66.2	368.6	529.2
Emergency room visits	83.0	272.6	71.1	308.1	59.3	248.9	59.3	248.9	47.4	225.2	320.0
Hospitalization	2741.1	5097.5	2452.6	5049.5	2308.3	5001.4	1875.5	4.953.3	1971.7	4.953.3	11,349.2
Medication	214.1	421.4	198.7	415.6	185.3	413.5	184.3	421.4	186.8	443.6	969.2
**Health-related cost**	**3561**	**5492**	**3123**	**5276**	**2910**	**5128**	**2447**	**4516**	**2493**	**4628**	**14.532.6**
**Indirect Cost/Sick Leave**	**33**	**423**	**34**	**455**	**41**	**564**	**45**	**593**	**42**	**599**	**195.8**
**Total Cost**	**3594**	**5915**	**3157**	**5731**	**2951**	**5691**	**2491**	**5109**	**2535**	**5227**	**14,728.4**

^a^In Euros

A specific analysis was performed in the DAPA-CKD like population (*n* = 5925). In this group of patients, mean age was 76.5 ± 14.6 years, 48.5% were women, and mean UACR was 420.7 ± 198.8 mg/g. Overall, 20.8% of patients had a history of HF, 13.4% MI, and 10.8% prior stroke. With regard to treatments, all patients were taking RAAS, but only 13.4% of patients at maximal doses. A total of 2951 (49.8%) patients had T2D. Patients with T2D had higher UACR (426.3 ± 201.5 vs 350.2 ± 171.4 mg/g; *P* < 0.001), and HbA1c (7.5 ± 2.0 vs 5.8 ± 1.3%; *P* < 0.001), but without significant differences in eGFR (49.5 ± 12.0 vs 50.0 ± 11.8 mL/min/1.73 m^2^; *P* = 0.336). In addition, comorbidities were more common among patients with T2D compared to those without T2D. Overall, in the DAPA-CKD like population, 95.2% had stage 3 or 4 CKD. UACR increased as renal function worsened (from 127.9 ± 58.5 in patients with stage 2 CKD to 1689.3 ± 841.3 mg/g among stage 4 CKD patients; *P* < 0.001), as well as comorbidities. In addition, the proportion of patients at maximal doses of ACEi or ARBs also increased as stage CKD worsened (from 10.3% in patients with stage 2 CKD to 17.2% among stage 4 CKD patients; *P* < 0.001) (supplementary Table 4).

With regard to patients hospital mean cost per year in the DAPA-CKD like population, there was a progressive decrease in cardiovascular disease hospital cost per year (from 3025.9 Euros in 2015 to 2022.9 Euros in 2019). Overall, patient cumulative cardiovascular disease hospital mean cost reached 12,219.0 Euros in 2019. The great burden of this cost was due to cardiovascular hospitalizations, particularly HF and CKD. Regarding medications, from 2015 to 2019, diabetes drugs mean cost decreased from 103.7 to 99.0 Euros and HF medication mean cost from 57.8 to 53.0 Euros, respectively. The cumulative mean cost of diabetes and HF medications reached 560.2 and 242.8 Euros, respectively, in 2019. Dialysis cost decreased from 2282.2 to 1591.7 Euros, respectively (cumulative cost of 9501.3 Euros) and kidney transplant from 727.8 to 502.4 Euros, respectively (cumulative cost of 2973.4 Euros) (supplementary Table 5).

## Discussion

Our data showed that in Spain, during the 2015–2019 period, CKD-associated costs were substantial, being cardiovascular hospitalizations the most important contributing factor (77.1%), mainly HF and CKD hospitalizations; however, medication cost contribution was marginal (6.6%). Of note, the annual cardiovascular hospitalization mean cost and mortality progressively decreased over time.

In our study, the prevalence of CKD was nearly 5% (mean age 76 years; 71% stage ≥3 CKD). Previously performed studies in Spain have shown a higher prevalence of CKD, possibly due to differences in the inclusion criteria, the methods for renal function determination and the study design. Since this is database study, patients with CKD risk factors (such as diabetes mellitus, hypertension or cardiovascular disease, who are not regularly screened and therefore identified) cannot be reflected, showing the high underdiagnosis rate of CKD still present nowadays. Additionally, this difference in CKD prevalence might be a consequence of a higher use of CKD protective treatments [23, 28–30]. Despite the fact that 71% of patients were taking RAAS inhibitors (82% among T2D patients) in our study, only 4.4% of patients reached maximal doses, suggesting that there is still a potential benefit on CKD outcomes with uptitration). It is likely that the risk of hyperkalemia or renal function impairment associated with these drugs, mainly in elderly patients or in advanced CKD could have had some impact on these results [31]. However, these numbers were not significantly higher in stage 1–2 CKD patients. As it has been reported that achieving maximal doses of RAAS inhibitors (vs lower doses) may be associated with better outcomes, it is highly recommended the use of cardiovascular and renal protective drugs at adequate doses to reduce outcomes [32, 33]. The reduction in the proportion of hospitalized patients, days of hospitalization and mortality during the 2015–2019 period could be related with a better comprehensive management of CKD population, including the use of guidelines recommended drugs [33–36].

More recently, the CREDENCE and DAPA-CKD trials have shown that among CKD patients with T2D, the use of SGLT-2 inhibitors translates into better cardiovascular and renal outcomes [9, 10]. In addition, the DAPA-CKD and the DECLARE-TIMI 58 trials have demonstrated that the beneficial effect of dapagliflozin on the development of cardiovascular and renal complications can be extended to the CKD population without T2D, and to T2D individuals with normal renal function at baseline, respectively [9, 34]. These data suggest that the addition of these drugs to the treatment of CKD patients could reduce even more morbidity and mortality, and consequently, overall CKD burden.

Our study showed that total cumulative cost of CKD patients was high. This has also been confirmed by previous studies [11–18]. The most important contributors for total health care cost in CKD patients were cardiovascular hospitalizations (admissions and hospital stay), particularly HF and CKD hospitalizations. This is not surprising, as CKD is associated with an increased risk of cardiovascular death, and progression to end-stage renal disease [37, 38]. There is a close relationship between CKD and HF. Thus, the presence of one condition promotes the development of the other, and vice versa [39]. In fact, HF can be an early complication of CKD. This has also been observed in the overall T2D population [39]. In the last years, a number of clinical trials have demonstrated the marked benefits of treatment with SGLT-2 inhibitors in the reduction of HF hospitalizations among T2D population [40]. Similarly, SGLT-2 inhibitors substantially decrease kidney composite outcomes in patients with T2D [41]. Unfortunately, in our study, only 2% of patients with T2D were taking SGLT-2 inhibitors at index date since this was 2015 and SGLT-2 inhibitors had been recently launched. It is very likely that the higher use of these drugs in T2D and non T2D populations will translate into a reduction of cardiovascular and renal complications and secondarily to a decrease of health care related costs [42, 43].

Different studies have shown that health care costs increase as renal function worsens or albuminuria develops, particularly in patients that finally require kidney replacement therapy [5, 11–14, 16, 20, 44–46]. As a result, although renal replacement therapy has been the object of constant analysis in order to improve the efficiency and sustainability, the fact is that preventing the occurrence and progression of CKD is the best way to reduce health care resource consumption and health care costs. Therefore, interventions designed to minimize decline in progressive kidney function, particularly among patients with stage 3 or 4 CKD, may reduce the economic CKD burden [5, 11–14, 16, 20, 44–47]. It has been reported that the addition of RAAS inhibitors to prevent the advance of nephropathy is worthwhile not only from a clinical perspective, but also from an economic point of view, even in patients with end stage renal disease, mainly driven by a reduction of hospitalization costs [47, 48]. Both, the CREDENCE and the DAPA-CKD trials showed that among CKD patients, the use of SGLT-2 inhibitors could prevent or delay the development of kidney complications, including end-stage renal disease [9, 10]. Our data showed a progressive reduction of costs associated with dialysis and kidney transplant. Although this is hopeful, a higher use of renal protective drugs, including RAAS inhibitors and SGLT-2 inhibitors with proven renal benefit, could provide additional benefits, including health care costs reduction.

Other contributors to total CKD cost included primary care visits, specialized visits, and diagnostic tests. It has been reported that not only the costs of specialized care decrease with the length of hospital stay reduction [19], but also a nephrologist/nurse-based multifaceted intervention for stage 3 to 4 CKD patients may be a cost effective approach [49], suggesting that an integrated management of CKD patients in both specialist and primary care settings is warranted to reduce CKD burden.

In our study, cardiovascular outcomes were more common in the DAPA-CKD like subpopulation than in the general CKD population [23], translating into higher costs. This has been confirmed in a real-world population similar to that of DAPA-CKD [50]. Despite the beneficial effects shown in the DAPA-CKD trial with dapagliflozin on the prevention of cardiovascular and renal outcomes in CKD patients, regardless the presence of T2D [9], in our study, less than 4% of T2D patients from the DAPA-CKD like subpopulation were taking SGLT-2 inhibitors [50]. As a result, it would be desirable a higher use of these drugs in this population with the double aim of decreasing outcomes and health care costs.

### Limitations

As this study had a retrospective design, only indirect causality may be suggested. In addition, some relevant data, such as albuminuria could not be documented in all patients, leading to an underdiagnosis of CKD. However, this is the best design to ascertain the therapeutic management of patients and health care costs in clinical practice, as no specific intervention was required to be included. Furthermore, the high number of patients included, as well as the robustness of the data allow achieving the objectives of the study. Unfortunately, medications were only recorded at baseline and no direct association can be determined between the decrease of events and costs and the use of cardiovascular medications. On the other hand, patients without a CKD diagnosis who met the definition of CKD stage 1 or higher were also considered as CKD patients and selected for the study. Although multiple readings of the eGFR are required to define CKD, due to the characteristics of the study, only one measurement was considered. However, the later represented only 6.9% of the total CKD study population and it was not expected that this had a significant impact on the results. Costs were taken from the Spanish National Healthcare System of 2019 and used for the overall study period. Although this could be a limitation, changes in costs during this time were marginal. In addition, improvements in efficiencies in hospital process may also reduce costs. Unfortunately, this could not be determined. Finally, although data came from seven Spanish regions, previous studies have shown that these data are representative of the entire Spanish population [21].

## Conclusions

During the period 2015–2019, costs of patients with CKD in Spain were substantial, with cardiovascular hospitalizations being the key determinant, particularly in HF with CKD. Medication costs were responsible for only a small proportion of total CKD costs. Costs and healthcare resources use were even higher in the DAPA-CKD like population. Improving CKD management, particularly with the use of cardiovascular and renal protective medications may be helpful to reduce CKD burden.

## Supplementary Information

**Additional file 1: Supplementary table 1.** Definition of variables. **Supplementary table 2.** Description of costs / units (year 2019). **Supplementary table 3.** Patient cumulative hospital mean cost*. **Supplementary table 4.** Baseline clinical characteristics of the DAPA-CKD population at index date (1st January 2015) and according to the presence of type 2 diabetes and CKD stage. **Supplementary table 5.** DAPA-CKD patients hospital mean cost for year and cumulative cost in 2019*.

## Data Availability

The data that support the findings of this study are available from BIG PAC® but restrictions apply to the availability of these data, which were used under license for the current study, and so are not publicly available. Data are however available from the authors upon reasonable request and with permission of BIG PAC®.

## Abbreviations

ACEi: Angiotensin-converting enzyme inhibitors
ARBs: Angiotensin-receptor blockers
AF: Atrial fibrillation
CKD: Chronic kidney disease
eGFR: estimated glomerular filtration rate
HF: Heart failure
MI: Myocardial infarction
PAD: Peripheral artery disease
RAAS: Renin angiotensin aldosterone system
SGLT-2: Sodium-glucose cotransporter-2
T2D: Type 2 diabetes
UACR: Urine albumin-to-creatinine ratio
