# Supplementary material for: Costs and healthcare utilisation of patients with chronic kidney disease in Spain
Source: BMC Health Serv Res. 2021 Jun 1;21:536. doi: 10.1186/s12913-021-06566-2 (PMC8167969; doi:10.1186/s12913-021-06566-2)
Supplement: Supplementary file 1 — Additional file 1: Supplementary table 1. Definition of variables. Supplementary table 2. Description of costs / units (year 2019). Supplementary table 3. Patient cumulative hospital mean cost*. Supplementary table 4. Baseline clinical characteristics of the DAPA-CKD population at index date (1st January 2015) and according to the presence of type 2 diabetes and CKD stage. Supplementary table 5. DAPA-CKD patients hospital mean cost for year and cumulative cost in 2019*. [file 12913_2021_6566_MOESM1_ESM.docx]

**Supplementary table 1**. **Definition of variables.**

| **Variable** | **Definition (codes)** |
| --- | --- |
| Myocardial infarction | I21, I22 |
| Stroke | I60-I63 |
| Heart failure | I50, I11.0, I13.0, I13.2 |
| CKD | N17-N19, I12.0-I2.9, I13.1, I13.2, N08.3, E10.2, E11.2, E12.2, E13.2, E14.2, Z49, Z99.2 + procedure codes |
| PAD | I70.2, I73.9, I74.2-9 |
| CVD related medications | B01AC06, C10AA, B01AA03, B01AC04, B01AC22, B01AC24, B01AC07, B01AC09, B01AC11, B01AC13, B01AC16, B01AC17, B01AC21 |
| HF related medications | C09A, C09B, C09C, C09D, C03C, C03DA, C07 |
| Diabetes related medications | All A10 medications |

CKD: chronic kidney disease; PAD: peripheral artery disease; CVD: cardiovascular disease; HF: heart failure.

**Supplementary table 2. Description of costs / units (year 2019)**

| Healthcare and non-healthcare resources | Unit costs (€) |
| --- | --- |
| Medical visits |  |
| Medical visits and primary care | 24,20 € |
| Emergency room visit | 118,50 € |
| Hospitalization (per day) | 480,90 € |
| Specialist medical visit | 94,50 € |
| Supplementary tests |  |
| Lab tests | 32,30 € |
| Conventional radiology | 28,50 € |
| Diagnostic test/therapeutic | 37,10 € |
| Computerized axial tomography | 96,00 € |
| Magnetic nuclear resonance | 177,00 € |
| Pharmaceutical prescription | RP+VAT |
| Work productivity - Indirect costs |  |
| Cost per day not worked | 101,21 € |
| RP: retail price. |  |

**Supplementary table 3. Patient cumulative hospital mean cost*.**

|  | **2015** | **2016** | **2017** | **2018** | **2019** |
| --- | --- | --- | --- | --- | --- |
| **Total hospital cost** | | | | | |
| CVD | 2,741.1 | 5,193.7 | 7,502.0 | 9,377.6 | 11,349.2 |
| Cardiorenal | 2,500.3 | 4,750.9 | 6,856.8 | 8,542.3 | 10,309.0 |
| HF | 1,514.3 | 2,856.2 | 4,140.2 | 5,255.1 | 6,267.7 |
| CKD | 986.0 | 1,894.7 | 2,716.7 | 3,287.1 | 4,041.3 |
| MI | 74.3 | 135.8 | 190.8 | 246.1 | 311.5 |
| Stroke | 111.7 | 211.1 | 316.5 | 411.1 | 510.9 |
| PAD | 54.9 | 95.9 | 137.8 | 178.0 | 217.8 |
| **Medication cost** | | | | | |
| Total medication cost | 181.8 | 360.6 | 541.6 | 684.3 | 852.0 |
| CVD and diabetic medication cost | 131.1 | 262.6 | 411.7 | 516.6 | 631.4 |
| Diabetes medication cost | 102.7 | 202.5 | 331.0 | 413.9 | 503.9 |
| HF medication cost | 50.7 | 98.0 | 129.9 | 167.6 | 220.6 |
| CVD medication cost | 28.4 | 60.1 | 80.7 | 102.8 | 127.5 |
| **Procedure cost** | | | | | |
| Total procedures costs | 2,984.5 | 5,642.0 | 8,158.5 | 10,215.3 | 12,304.7 |
| Dialysis | 2,328.8 | 4,410.1 | 6,375.3 | 7,978.7 | 9,602.9 |
| Kidney transplant | 655.7 | 1,231.9 | 1,783.2 | 2,236.6 | 2,701.8 |

*In Euros. Cardiorenal costs include HF and CKD cost.

CVD: cardiovascular disease; HF: heart failure; CKD: chronic kidney disease; MI: myocardial infarction; PAD: peripheral artery disease.

**Supplementary table 4. Baseline clinical characteristics of the DAPA-CKD population at index date (1^st^ January 2015) and according to the presence of type 2 diabetes and CKD stage**.

|  | **Diabetes status** | | | **CKD stage** | | | | | | | | | | **Total (n=5,925; 100%)** |
| --- | --- | --- | --- | --- | --- | --- | --- | --- | --- | --- | --- | --- | --- | --- |
|  | **Non T2D (n=2,974; 50.2%)** | **T2D (n=2,951; 49.8%)** | **p** | **Stage 1 (n=; 0%)** | **Stage 2 (n=284; 4.8%)** | **Stage 3a (n=2,673; 45.1%)** | **P_3a vs 2_** | **Stage 3b (n=2,263; 38.2%)** | **P_3b vs 2_** | **Stage 4 (n=705; 11.9%)** | **P_4vs 2_** | **Stage 5 (n=0; 0%)** | **Unspecified (n=0; 0%)** |  |
| **Biodemographic data** | | | | | | | | | | | | | | |
| Age, years | 76.2±14.2 | 76.8±14.2 | 0.001 | NA | 77.2±14.4 | 77.0±14.2 | 0.423 | 79.8±14.1 | <0.001 | 79.9±14.6 | <0.001 | NA | NA | 76,5±14.6 |
| Sex. Female, n (%) | 1,478 (49.7) | 1,394 (47.2) | 0.054 | NA | 137 (48.2) | 1,270 (47.5) | 0.822 | 1,129 (49.9) | 0.589 | 336 (47.7) | 0.887 | NA | NA | 2,872 (48,5) |
| **Physical examination and laboratory tests** | | | | | | | | | | | | | | |
| SBP, mmHg | 136.2±20.2 | 136.7±20.2 | 0.336 | NA | 138.1±20.7 | 137.8±20.6 | 0.810 | 138.9±20.4 |  | 136.6±20.5 |  | NA | NA | 136.4±20.2 |
| UACR, mg/g | 350.2±171.4 | 426.3±201.5 | <0.001 | NA | 127.9±58.5 | 253.2±116.4 | <0.001 | 260.2±122.7 | <0.001 | 1,689.3±841.3 | <0.001 | NA | NA | 420.7±198.8 |
| UACR A1 | 0 | 0 | -- | NA | 0 | 0 | -- | 0 | -- | 0 | -- | NA | NA | 0 |
| UACR A2 | 1,929 (64.9) | 1,870 (63.4) | 0.229 | NA | 284 (100) | 1,759 (65.8) | -- | 1,491 (65.9) | -- | 265 (37.6) | -- | NA | NA | 3,799 (64.1) |
| UACR A3 | 1,045 (35.1) | 1,081 (36.6) | 0.229 | NA | 0 | 914 (34.2) | -- | 772 (34.1) | -- | 440 (62.4) | -- | NA | NA | 2,126 (35.9) |
| eGFR* | 50.0±11.8 | 49.5±12.0 | 0.336 | NA | 75.0±5.0 | 51.9±5.1 | <0.001 | 37.1±5.0 | <0.001 | 22.2±5.1 | <0.001 | NA | NA | 49.7±13.3 |
| eGFR ≥90*,n (%) | 0 | 0 | -- | NA | 0 | 0 | -- | 0 | -- | 0 | -- | NA | NA | 0 |
| eGFR 60-89*, n (%) | 163 (5.5) | 121 (4.1) | 0.012 | NA | 284 (100) | 0 | -- | 0 | -- | 0 | -- | NA | NA | 284 (4.8) |
| eGFR 45-59*, n (%) | 1,442 (48.5) | 1,231 (41.7) | <0.001 | NA | 0 | 2,673 (100) | -- | 0 | -- | 0 | -- | NA | NA | 2,673 (45.1) |
| eGFR 30-44*, n (%) | 1,065 (35.8) | 1,198 (40.6) | <0.001 | NA | 0 | 0 | -- | 2,263 (100) | -- | 0 | -- | NA | NA | 2,263 (38.2) |
| eGFR 15-29*, n (%) | 304 (10.2) | 401 (13.6) | 0.001 | NA | 0 | 0 | -- | 0 | -- | 705 (100) | -- | NA | NA | 705 (11.9) |
| eGFR <15*, n (%) | 0 | 0 | - | NA | 0 | 0 | -- | 0 | -- | 0 | -- | NA | NA | 0 |
| HbA1c. % | 5.8±1.3 | 7.5±2.0 | <0.001 | NA | 6.5±1.5 | 6.6±1.5 | 0.949 | 6.7±1.6 | 0.899 | 7.2±1.7 | 0.696 | NA | NA | 6.6±1.9 |
| Creatinine. g/dL | 1.1±0.4 | 1.2±0.5 | <0.001 | NA | 0.9±0.4 | 1.3±0.5 | <0.001 | 1.7±0.4 | 0.313 | 2.3±0.9 | 0.144 | NA | NA | 1.1±0.6 |
| Uric acid. g/dL | 5.8±1.8 | 7.0±1.1 | <0.001 | NA | 6.9±1.8 | 6.8±1.7 | 0.949 | 6.4±1.5 | 0.747 | 7.1±1.6 | 0.911 | NA | NA | 6.4±1.7 |
| **Comorbidities, n (%)** | | | | | | | | | | | | | | |
| CKD stage 1 | 0 | 0 |  | NA | 0 | 0 | -- | 0 | -- | 0 | -- | NA | NA | 0 |
| CKD stage 2 | 163 (5.5) | 121 (4.1) | 0.010 | NA | 284 (100) | 0 | -- | 0 | -- | 0 | -- | NA | NA | 284 (4.8) |
| CKD stage 3a | 1,442 (48.5) | 1,231 (41.7) | <0.001 | NA | 0 | 2,673 (100) | -- | 0 | -- | 0 | -- | NA | NA | 2,673 (45.1) |
| CKD stage 3b | 1,065 (35.8) | 1,198 (40.6) | <0.001 | NA | 0 | 0 | -- | 2,263 (100) | -- | 0 | -- | NA | NA | 2,263 (38.2) |
| CKD stage 4 | 304 (10.2) | 401 (13.6) | 0.001 | NA | 0 | 0 | -- | 0 | -- | 705 (100) | -- | NA | NA | 705 (11.9) |
| CKD stage 5 | 0 | 0 | -- | NA | 0 | 0 | -- | 0 | -- | 0 | -- | NA | NA | 0 |
| CKD not staged | 0 | 0 | -- | NA | 0 | 0 | -- | 0 | -- | 0 | -- | NA | NA | 0 |
| CKD unspecified | 882 (29.7) | 167 (5.7) | <0.001 | NA | 43 (15.1) | 489 (18.3) | 0.182 | 403 (17.8) | 0.259 | 114 (16.2) | 0.669 | NA | NA | 1,049 (17.7) |
| Dialysis | 0 | 0 | -- | NA | 0 | 0 | -- | 0 | -- | 0 | -- | NA | NA | 0 |
| CVD | 501 (16.9) | 603 (20.4) | 0.001 | NA | 46 (16.3) | 515 (19.3) | 0.221 | 429 (19.0) | 0.271 | 114 (16.2) | 0.969 | NA | NA | 1,104 (18.6) |
| Myocardial infarction | 343 (11.6) | 451 (15.3) | 0.001 | NA | 34 (12.0) | 344 (12.9) | 0.662 | 308 (13.6) | 0.456 | 108 (15.3) | 0.181 | NA | NA | 794 (13.4) |
| Heart failure | 555 (18.7) | 679 (23.0) | <0.001 | NA | 39 (13.8) | 536 (20.0) | 0.012 | 494 (21.8) | 0.002 | 165 (23.4) | 0.001 | NA | NA | 1,234 (20.8) |
| Stroke | 306 (10.3) | 331 (11.2) | 0.263 | NA | 25 (8.9) | 262 (9.8) | 0.626 | 279 (12.3) | 0.096 | 71 (10.1) | 0.565 | NA | NA | 637 (10.8) |
| Atrial Fibrillation | 499 (16.8) | 524 (17.8) | 0.309 | NA | 39 (13.7) | 464 (17.4) | 0.115 | 399 (17.6) | 0.100 | 121 (17.2) | 0.177 | NA | NA | 1,023 (17.3) |
| Peripheral artery disease | 122 (4.1) | 184 (6.2) | <0.001 | NA | 14 (5.1) | 132 (4.9) | 0.882 | 118 (5.2) | 0.943 | 42 (5.9) | 0.623 | NA | NA | 306 (5.2) |
| Diabetes | 166 (5.6) | 2,951 (100) | <0.001 | NA | 140 (49.3) | 1,298 (48.6) | 0.882 | 1,232 (54.4) | 0.104 | 447 (63.4) | <0.001 | NA | NA | 3,117 (52.6) |
| **Medications, n (%)** | | | | | | | | | | | | | | |
| **Antihypertensives** | 2,186 (73.5) | 2,526 (85.6) | <0.001 | NA | 222 (78.2) | 2,061 (77.1) | 0.674 | 1,826 (80.7) | 0.317 | 603 (85.5) | 0.005 | NA | NA | 4,712 (79.5) |
| RAAS inhibitors | 2,974 (100) | 2,951 (100) | - | NA | 284 (100) | 2,673 (100) | -- | 2,263 (100) | -- | 705 (100) | -- | NA | NA | 5,925 (100) |
| ACEi | 1,247 (41.9) | 1,202 (40.7) | 0.348 | NA | 92 (32.4) | 983 (36.8) | 0.143 | 1,022 (45.2) | <0.001 | 353 (50.0) | 0.001 | NA | NA | 2,449 (41.3) |
| ACEi at maximal doses | 178 (6.0) | 169 (5.7) | 0.629 | NA | 13 (4.5) | 148 (5.5) | 0.479 | 136 (6.0) | 0.309 | 51 (7.3) | 0.106 | NA | NA | 347 (5.9) |
| ARBs | 1,704 (57.3) | 1,770 (60.0) | 0.035 | NA | 131 (46.0) | 1,400 (52.4) | 0.040 | 1,307 (57.8) | <0.001 | 636 (90.2) | <0.001 | NA | NA | 3,474 (58.6) |
| ARBs at maximal doses | 216 (7.3) | 227 (7.7) | 0.559 | NA | 16 (5.8) | 170 (6.4) | 0.693 | 188 (8.3) | 0.144 | 69 (9.9) | 0.039 | NA | NA | 443 (7.5) |
| MRAs | 152 (5.1) | 258 (8.7) | <0.001 | NA | 15 (5.1) | 169 (6.3) | 0.425 | 157 (6.9) | 0.253 | 69 (9.8) | 0.016 | NA | NA | 410 (6.9) |
| Direct renin inhibitors | 26 (0.9) | 68 (2.3) | <0.001 | NA | 3 (1.2) | 40 (1.5) | 0.690 | 38 (1.7) | 0.532 | 12 (1.8) | 0.500 | NA | NA | 94 (1.6) |
| ARNI | 186 (6.3) | 213 (7.2) | 0.126 | NA | 14 (5.0) | 158 (5.9) | 0.538 | 161 (7.1) | 0.187 | 66 (9.4) | 0.022 | NA | NA | 399 (6.7) |
| Beta blockers | 957 (32.2) | 1,275 (43.2) | <0.001 | NA | 83 (29.4) | 952 (35.6) | 0.037 | 916 (40.5) | <0.001 | 280 (39.8) | 0.002 | NA | NA | 2,232 (37.7) |
| Diuretics | 1,095 (36.8) | 1,318 (44.7) | <0.001 | NA | 84 (29.5) | 950 (35.6) | 0.041 | 993 (43.9) | <0.001 | 386 (54.8) | 0.001 | NA | NA | 2,413 (40.7) |
| Thiazide diuretics | 128 (4.3) | 144 (4.9) | 0.270 | NA | 11 (3.8) | 98 (3.7) | 0.932 | 110 (4.9) | 0.413 | 53 (7.6) | 0.028 | NA | NA | 272 (4.6) |
| Loop diuretics | 979 (32.9) | 1,165 (39.5) | <0.001 | NA | 74 (26.2) | 893 (33.4) | 0.014 | 863 (38.1) | <0.001 | 314 (44.5) | <0.001 | NA | NA | 2,144 (36.2) |
| Potassium sparing diuretics | 217 (7.3) | 249 (8.4) | 0.115 | NA | 18 (6.5) | 181 (6.8) | 0.848 | 180 (8.0) | 0.375 | 87 (12.3) | 0.007 | NA | NA | 466 (7.9) |
| CCB | 836 (28.1) | 1,105 (37.4) | <0.001 | NA | 74 (26.2) | 757 (28.3) | 0.454 | 817 (36.1) | 0.001 | 292 (41.4) | <0.001 | NA | NA | 1,941 (32.8) |
| Dihydropyridines | 749 (25.2) | 1,035 (35.1) | <0.001 | NA | 68 (23.9) | 704 (26.3) | 0.381 | 744 (32.9) | 0.002 | 268 (38.1) | <0.001 | NA | NA | 1,784 (30.1) |
| Non- dihydropyridines | 113 (3.8) | 95 (3.2) | 0.209 | NA | 7 (2.6) | 80 (3.0) | 0.705 | 85 (3.8) | 0.310 | 35 (5.0) | 0.093 | NA | NA | 208 (3.5) |
| **Antidiabetics** | 83 (2.8) | 2,349 (79.6) | <0.001 | NA | 97 (34.2) | 985 (36.8) | 0.387 | 1,024 (45.2) | <0.001 | 326 (46.2) | 0.001 | NA | NA | 2,432 (41.0) |
| Metformin | 0 | 1,384 (46.9) | <0.001 | NA | 45 (15.8) | 576 (21.5) | 0.025 | 587 (25.9) | <0.001 | 176 (25.0) | 0.002 | NA | NA | 1,384 (23.4) |
| Sulfonylurea | 0 | 394 (13.4) | <0.001 | NA | 13 (4.6) | 162 (6.1) | 0.310 | 166 (7.3) | 0.093 | 53 (7.5) | 0.098 | NA | NA | 394 (6.7) |
| DPP4 inhibitors | 0 | 1,105 (37.4) | <0.001 | NA | 42 (14.8) | 443 (16.6) | 0.436 | 426 (18.8) | 0.101 | 194 (27.5) | <0.001 | NA | NA | 1,105 (18.7) |
| SGLT-2 inhibitors | 0 | 115 (3.9) | <0.001 | NA | 4 (1.5) | 55 (2.1) | 0.497 | 46 (2.1) | 0.500 | 9 (1.3) | 0.806 | NA | NA | 115 (1.9) |
| GLP-1 receptor agonists | 0 | 99 (3.3) | <0.001 | NA | 4 (1.6) | 34 (1.3) | 0.675 | 36 (1.6) | 0.999 | 24 (3.4) | 0.126 | NA | NA | 99 (1.7) |
| Metiglinides | 0 | 388 (13.1) | <0.001 | NA | 14 (5.0) | 167 (6.3) | 0.387 | 176 (7.8) | 0.091 | 30 (4.3) | 0.631 | NA | NA | 388 (6.5) |
| Glitazones | 0 | 89 (3.0) | <0.001 | NA | 3 (1.1) | 940 (1.5) | 0.593 | 29 (1.3) | 0.777 | 18 (2.5) | 0.165 | NA | NA | 89 (1.5) |
| Acarbose | 0 | 70 (2.4) | 0.001 | NA | 5 (1.7) | 925 (0.9) | 0.193 | 27 (1.2) | 0.476 | 13 (1.8) | 0.914 | NA | NA | 70 (1.2) |
| Insulin | 83 (2.8) | 597 (20.2) | <0.001 | NA | 25 (8.8) | 281 (10.5) | 0.371 | 252 (11.1) | 0.240 | 122 (17.4) | 0.001 | NA | NA | 680 (11.5) |
| Low dose aspirin | 782 (26.3) | 806 (27.3) | 0.385 | NA | 58 (20.4) | 651 (24.4) | 0.134 | 656 (29.0) | 0.002 | 222 (31.5) | 0.001 | NA | NA | 1,588 (26.8) |
| Statins | 1,389 (46.7) | 1,864 (63.1) | <0.001 | NA | 107 (37.5) | 1,400 (52.4) | <0.001 | 1,278 (56.5) | <0.001 | 468 (66.4) | <0.001 | NA | NA | 3,253 (54.9) |
| Warfarin | 397 (13,3) | 413 (14.0) | 0.433 | NA | 34 (11.9) | 317 (11.9) | 0.999 | 334 (14.8) | 0.191 | 125 (17.7) | 0.025 | NA | NA | 810 (13.7) |
| Receptor P2Y12 antagonists | 49 (1,7) | 45 (1.5) | 0.540 | NA | 2 (0.8) | 34 (1.3) | 0.471 | 41 (1.8) | 0.218 | 17 (2.4) | 0.099 | NA | NA | 94 (1.6) |

ACEi: angiotensin-converting enzyme inhibitors; ARBs: angiotensin receptor blockers; ARNI: angiotensin receptor and neprilysin inhibition; BMI: body mass index; CCB: Calcium channel blockers; CVD: cardiovascular disease; ; CKD: chronic kidney disease; DPP4: dipeptidyl peptidase 4; eGFR: estimated glomerular filtration rate; * mL/min/1.73 m^2^; GLP-1: glucagon-like peptide-1; PAD: peripheral artery disease; RAAS: renin angiotensin system; SBP: systolic blood pressure; SGLT-2: sodium-glucose Cotransporter-2; UACR: Urine albumin-to-Creatinine Ratio.

**Supplementary table 5. DAPA-CKD patients hospital mean cost for year and cumulative cost in 2019*.**

|  | **2015** | | **2016** | | **2017** | | **2018** | | **2019** | | **Cumulative cost in 2019** |
| --- | --- | --- | --- | --- | --- | --- | --- | --- | --- | --- | --- |
|  | mean | SD | mean | SD | mean | SD | mean | SD | mean | SD |  |
| **Total hospital cost** | | | | | | | | | | | |
| CVD | 3,025.9 | 6,214.3 | 2,822.1 | 6,023.5 | 2,348.6 | 5,623.1 | 1,999.6 | 5,921.2 | 2,022.9 | 5,023.4 | 12,219.0 |
| Cardiorenal | 2,763.0 | 5,823.2 | 2,593.4 | 5,498.1 | 2,145.4 | 4,963.7 | 1,786.8 | 3,658.8 | 1.804,9 | 3,824.3 | 11,093.6 |
| HF | 1,786.9 | 3,458.3 | 1,503.0 | 3,444.9 | 1,348.1 | 3,114.6 | 1,170.7 | 2,520.9 | 1,073.3 | 2,620.8 | 6.882,0 |
| CKD | 976.1 | 4,044.9 | 1,090.5 | 4,106.7 | 797.3 | 3,860.2 | 616.1 | 3,353.9 | 731.6 | 3,479.1 | 4,211.6 |
| MI | 73.5 | 696.1 | 70.2 | 571.5 | 54.5 | 526.4 | 58.1 | 537.8 | 67.4 | 703.8 | 323.6 |
| Stroke | 129.6 | 784.8 | 111.3 | 814.5 | 105.4 | 895.1 | 106.9 | 819.8 | 103.8 | 741.5 | 557.0 |
| PAD | 59.8 | 691.8 | 47.1 | 572.6 | 43.2 | 620.9 | 47.8 | 558.0 | 46.9 | 563.0 | 244.9 |
| **Medication cost** | | | | | | | | | | | |
| Total medication | 191.1 | 398.2 | 202.9 | 430.4 | 208.4 | 352.3 | 161.5 | 251.8 | 176.3 | 348.1 | 940.1 |
| Diabetes medication | 103.7 | 337.7 | 109.8 | 348.0 | 149.1 | 514.6 | 98.6 | 317.9 | 99.0 | 344.0 | 560.2 |
| HF medication | 57.8 | 85.2 | 55.4 | 81.1 | 36.7 | 50.5 | 40.0 | 64.0 | 53.0 | 100.9 | 242.8 |
| CVD medication | 29.5 | 70.5 | 37.7 | 70.2 | 22.6 | 48.0 | 23.0 | 53.3 | 24.2 | 53.0 | 137.1 |
| **Procedure cost** | | | | | | | | | | | |
| Total procedures | 3,010.0 | 26,657.2 | 2,733.7 | 23,364.1 | 2,571.6 | 22,067.9 | 2,065.2 | 16,867.4 | 2,094.1 | 17,325.6 | 3,010.0 |
| Dialysis | 2,282.2 | 23,342.2 | 2,122.9 | 21,245.9 | 1,965.2 | 19,634.7 | 1,539.3 | 17,860.9 | 1,591.7 | 17,321.7 | 9,501.3 |
| Kidney | 727.8 | 5,665.1 | 610.8 | 4,267.9 | 606.4 | 4,288.1 | 525.9 | 3,593.8 | 502.4 | 3,402.9 | 2,973.4 |

*In Euros.

CVD: cardiovascular disease; HF: heart failure; CKD: chronic kidney disease; MI: myocardial infarction; PAD: peripheral artery disease.

Cardiorenal costs include HF and CKD cost.
